# Youth Sensitivity in a Pandemic: The Relationship Between Sensory Processing Sensitivity, Internalizing Problems, COVID-19 and Parenting

**DOI:** 10.1007/s10826-022-02243-y

**Published:** 2022-04-07

**Authors:** Selina S. C. Burgard, Juliëtte M. Liber, Suzanne M. Geurts, Ina M. Koning

**Affiliations:** 1 Department of Developmental Psychology, Utrecht University, Heidelberglaan 1, 3584 CS Utrecht, Netherlands; 2 PsyMens, Kind en Jeugd, Korenmolenlaan 1d, 3447 GG Woerden, Netherlands; 3 Department of Interdisciplinary Social Science, Utrecht University, Heidelberglaan 8, 3584 CS Utrecht, Netherlands

**Keywords:** Sensory processing sensitivity, Internalizing problems, Adolescence, Parenting style, COVID 19

## Abstract

The personality trait sensory processing sensitivity (SPS) is an established risk factor for the development of internalizing problems. Highly sensitive adolescents react stronger to environmental cues including parenting environment and stressful life events. The aim of the current study was to examine if the perceived impact of COVID-19, mediates the link between SPS and internalizing problems. In addition, it was tested if parenting style moderates the mediating effect of perceived COVID-19 impact between SPS and internalizing problems among adolescents. The study had a cross- sectional design and data were collected between April-July 2020 during the first lockdown in the Netherlands. Participants were 404 adolescents aged 9–18 years (M_age_ = 13.49). Questionnaires were administered online to assess SPS (Highly Sensitive Child Scale), parenting style (Parenting Style Inventory-II), internalizing problems (Patient Health Questionnaire-4) and COVID-19 pandemic impact (COVID-19 impact scale). The SPSS macro PROCESS was used to test the mediation model of perceived COVID-19 impact and the moderated mediation model with parenting style as a moderator. A relationship was found between SPS and internalizing problems which is partly mediated by the COVID-19 impact. The moderating effect of parenting style was not found. These findings provide insight into the effect the pandemic has had on highly sensitive adolescents. Further research is needed to develop and test interventions to support sensitive youth and thus possibly prevent the development of internalizing problems.

## Highlights

- The relationship between sensory processing sensitivity (SPS) and internalizing problems in adolescents is partly mediated by the perceived COVID-19 impact (stressful life event).
- Parenting style has no moderating effect on the relationship between SPS and internalizing problems.
- Permissive parenting is related to less internalizing problems and less perceived negative COVID-19 impact.

In the first months of 2020, the SARS-CoV-2 virus (also known as Coronavirus or COVID-19) spread rapidly around the world resulting in a once-in-a-century pandemic (Gates, 2020). In response to rising infection numbers, the Dutch government adopted a set of safety measures labelled as an “intelligent lockdown” implemented as of March 16^th^, 2020. These measures included the closure of public places (e.g., schools, cafes, and museums), instructions to stay at home and keep social distance, and quarantine in the case of infection. The virus, as well as the intelligent lockdown, have led to many changes in people’s daily lives, including the life of adolescents, on a scale that is unprecedented in modern history, posing a risk to adolescents’ development as well as emotional and psychological wellbeing (Dawson & Golijani-Moghaddam, 2020; Prime et al., 2020). Preliminary research has already shown increased levels of stress, anxiety, and depressive symptoms in children and adolescents during the COVID-19 pandemic (Brooks et al., 2020; Dawson & Golijani-Moghaddam, 2020; Orgilés et al., 2020; Xie et al., 2020).

Anxiety, depression, and stress can be summarized under the broader category of internalizing problems, defined as occurring within a person rather than being acted out externally in the environment (Graber & Sontag, 2009). Internalizing problems during childhood and adolescence are predictive of multiple negative developmental outcomes such as peer victimization (Reijntjes et al., 2010), internalizing disorders (e.g., anxiety, depression), and poor health later in life (Essex et al., 2014; Essex et al., 2009; Herrenkohl et al., 2010). Given the severity of the possible negative developmental outcomes of internalizing problems, it is important to determine risk factors predictive of internalizing problems during childhood and adolescence, particularly in times of COVID-19.

Research has identified the personality trait sensory processing sensitivity (SPS) as a risk factor for developing internalizing problems (Aron et al., 2012; Slagt et al., 2018). SPS is a relatively stable trait that reflects an individual’s sensitivity to environmental influences such as other people’s expressed emotions, loud noises and pain, and the intensity of the individuals reaction in response. Higher levels of SPS are associated with a feeling of overstimulation in response to excessive demands. Although there is considerable overlap with the concept of temperamental reactivity, SPS draws on the literature on personality traits. A high score on the SPS trait is related to internalizing problems like depression or anxiety symptoms (Aron et al., 2005; Bakker & Moulding, 2012; Boterberg & Warreyn, 2016; Dal, 2016; Evers et al., 2008; Liss et al., 2008; Liss et al., 2005). Also, a study of Dean et al., (2018) showed among a sample of typically-developing children that a higher level of SPS related to more externalizing and internalizing problems. Little is known about the mechanisms underlying the impact of SPS on adolescents’ internalizing problems. The peculiar situation of the COVID-19 pandemic, which was experienced as stressful by some, may be a potential mediator in the relation between SPS and internalizing problems.

In this study, we want to examine perceived COVID-19 impact as a mediator of the link between SPS and internalizing problems. According to Baron & Kenny (1986), a mediator is defined as a variable that changes as a result of the predictor and subsequently affects a third variable. Thus, the current study will examine whether the COVID-19 impact adolescents perceive changes as a result of their level of SPS and subsequently affects the development of internalizing problems. Recent research on the impact of the COVID-19 pandemic as a stressful life event reveals a rise in internalizing problems in youth (Brooks et al., 2020; Dawson & Golijani-Moghaddam, 2020). The relationship between stressful life events and internalizing problems has been well established (e.g. Graber & Sontag 2009). A study on the mental health status of Chinese children reported more than usual depression and anxiety symptoms during the lockdown (Xie et al., 2020). In a similar study in Italy and Spain, 85.7% of the parents reported changes in their children’s emotional state and behaviors during the quarantine (Orgilés et al., 2020). Thus, these findings strongly suggest that the COVID-19 impact is related to an increase in internalizing problems in youth across nations.

Obviously, a stressful life event itself (here: COVID-19 pandemic) does not increase because of an individual’s characteristic (here: SPS). However, a person’s perception of the impact of a stressful life event can vary depending on individual characteristics. High SPS individuals show a stronger emotional reactivity to the environmental context (Aron & Aron, 1997; Aron et al., 2012). Even though the COVID-19 pandemic is a stressful life event on a community level, the perceived impact of the situation varies greatly from person to person. Individuals with high SPS scores are more shaken than others by changes in their life (Aron et al., 2012). In line with the trait-by-environment design of the diathesis-stress model (trait x environment) it can be expected that highly sensitive youth perceive a stronger negative impact by the COVID-19 pandemic which leads to more internalizing problems. Earlier research has identified the perceived impact of stressful life events as a possible mediator between risk factors and internalizing problems (Luby et al., 2006). Stressful life events can be defined as occurrences in a person’s life that change the usual activities and require considerable readjustment (Dohrenwend 2006). A stressful life event can occur on a personal level, like the loss of a loved one, or affect an entire community, like an earthquake (Schwarzer & Luszczynska, 2012). The COVID-19-pandemic and the resulting lockdown situation constitute drastic changes in the daily lives of adolescents, including a sudden switch to online education, not being able to participate in sports and deprivation of real-life peer interactions. The pandemic can therefore be categorized as a potential stressful life event for adolescents. Therefore, adolescents with a higher level of SPS may be more sensitive to stressful contexts, such as the lockdown during the COVID-19 situation and may therefore experience more internalizing problems.

To obtain more insight into the relation between SPS and internalizing problems via impact of COVID-19, highly sensitive individuals should be considered in the context of their environment. During the lockdown, adolescents had to spend a large amount of time at home under the care of and in close proximity to their parents. This makes parenting an even more influential and challenging environmental factor that may moderate the relation between SPS, perceived COVID-19 impact and internalizing problems in adolescents.

Parenting practices interact with the personality of a child in predicting behavioral outcomes (Pluess & Belsky, 2010). According to the differential susceptibility model (Belsky & Pluess, 2016), certain susceptibility factors promote individuals to be more influenced by both positive and negative environmental stimuli when compared to others without those traits (Rabinowitz & Drabick, 2017). SPS is such a susceptibility factor. Adolescents scoring high on the SPS personality trait appear more susceptible to environmental influences in a for-better-and-for-worse manner, resulting in worse developmental outcomes under negative circumstances but also better developmental outcomes in a supportive environment (Belsky & Pluess, 2016). Adolescents high in SPS respond to environmental cues with stronger emotional reactivity and are therefore more impacted by the influences of the parenting environment (Aron & Aron, 1997). Highly sensitive children experienced more negative affectivity including depression in the context of a less caring parental environment (Aron et al., 2005). The relationship between high SPS and internalizing problems is moderated by parental care indicating that highly sensitive adolescents might be particularly impacted by uncaring parents (Liss et al., 2008). Earlier research has also found an interaction between SPS and negative parenting styles in predicting indices of psychopathology (Sadoughi et al., 2007). These findings provide evidence that parenting can have a moderating effect on the relationship between SPS and internalizing problems in adolescents.

A common way to measure parenting is through parenting dimensions and the subsequent parenting styles, of which responsiveness and demandingness are considered the main dimensions. Responsiveness and demandingness of parenting are linked to child well-being not just in isolation, but also in the way they interact to describe patterns of parenting (Power, 2013). The first to use parenting dimensions to categorize distinct parenting styles was Baumrind (1967, 1971). Building on that theory, Maccoby & Martin (1983) described parents in terms of their position on the two main parenting dimensions, responsiveness and demandingness. The four parenting styles emerging from this framework are (1) authoritative (high in responsiveness and demandingness); (2) authoritarian (low in responsiveness and high in demandingness), (3) permissive (high in responsiveness and low in demandingness), and (4) neglectful (low in responsiveness and demandingness; Power 2013). Authoritative parenting is suggested to be optimal for developmental outcomes whereas authoritarian, permissive, and neglectful parenting styles have been linked to poorer outcomes for children and adolescents (Newman et al., 2008; Pinquart, 2017). Thus, a parenting style that is characterized by high responsiveness and demandingness is considered most optimal as to a variety of adolescent developmental outcomes.

Furthermore, parenting style may interact with SPS in predicting the COVID-19 impact perceived by adolescents. There is some evidence that parenting style can have a moderating effect on the impact certain stressful life events have on children (Slone et al., 2012). The COVID-19 induced lockdown is a stressful life event which coincides with adolescents spending more time at home under the care of their parents. It is therefore expected that the parenting style will affect the impact that the pandemic has on adolescents. While an authoritative parenting style can act as a protecting factor, the other three parenting styles might worsen the perceived negative impact of this stressful life event among adolescents high in SPS.

**Fig. 1:**
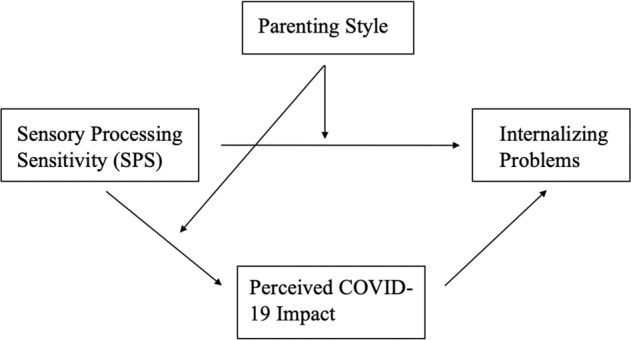
The proposed moderated mediation model

While earlier research established the relationship between SPS characteristics and internalizing problems in children and adolescents, the moderating effect of parenting styles has not yet been examined. Furthermore, no research has explored the above-mentioned constructs in the context of a stressful life event like the COVID-19 pandemic yet, as far as the authors are aware of. The goal of the current study was to address the knowledge gap in the literature, and investigate the relations among SPS, perceived COVID-19 impact and internalizing problems in adolescents, and moderation by parenting styles. A positive relation between SPS and internalizing problems is expected, and this relation is hypothesized to be (partially) mediated via perceived COVID-19 impact. Furthermore, it is expected that an authoritative parenting style is protective in the impact of SPS on internalizing problems as well as on the complete mediation relations. Data collection among adolescents during the lockdown period provides a unique possibility to examine the proposed moderated mediation model (Fig. 1).

## Methods

### Participants

The total sample consisted of 404 children and adolescents attending primary or secondary school. The initial sample was reduced in two ways. First, all participants who were older than 18 were removed from the sample (*n* = 1). Then, participants with missing data were removed (*n* = 8). The final sample consisted of 395 participants aged 9 to 18 years (*M* = 13.49, *SD* = 2.15). Forty-six percent of the participants were male. Most youth (68.6%) attended secondary school at the time the data were collected (typical age range 11 to 18 years). The sample was predominantly Dutch (96.5%).

### Procedure

Our study had a cross-sectional design and examined between-person differences. Adolescent self-report questionnaires from the first measurement wave of the Digital Family project were used. The Digital Family project is an ongoing Dutch longitudinal study primarily investigating youth digital media use in the context of the family. Participants were recruited through different channels, like social media and personal networks. Besides, schools that successfully participated in a previous study were asked to include the recruitment information in their newsletter. The data collection was conducted in April-July 2020, which coincided with the “intelligent lockdown” implemented by the Dutch government to slow the exponential spread of the COVID-19 virus. This entailed the closure of all childcare institutions, schools, sport clubs and foodservice industry as well as the stimulation of social distancing by staying home as much as possible. All participants who signed up for the study received an email with the link to an online questionnaire. Before starting the questionnaire, participants were presented with an informed consent form, which disclosed to them that the data would be used unanimously and that they could stop participating in the study at any time. Parents provided active consent for children aged <16 years. After accepting the informed consent form, participants could start completing the online questionnaires which took about 25 min to complete. They were asked to answer the questions honestly. For completing the questionnaire, participants received a 5 Euro gift-voucher. Data collection was approved by the Ethics Committee of the Faculty of Social and Behavioral Science at Utrecht University (FETC20-192).

### Instruments

*Sensory Processing Sensitivity* (SPS) was measured by a shortened version of the Highly Sensitive Child Scale (HSC; Pluess et al., 2018). The original scale used twelve items to determine the overall sensitivity with 3 items specifically measuring the subcategory low sensory threshold (LST), four items measuring the subcategory aesthetic sensitivity (AES), and five items measuring the subcategory ease of excitation (EOE). The AES subcategory was excluded since recent research shows that AES neither sufficiently correlates with the other two subcategories nor with the negative outcomes associated with SPS (Ershova et al., 2018; Liss et al., 2008). Examples for items measuring the remaining two subcategories are ‘Loud noises make me uncomfortable’ (LST) or ‘I don’t like change’ (EOE). Participants could answer on a 5-point Likert scale (1 = I don’t agree at all, 5 = I completely agree). The variable SPS can be operationalized as a categorical as well as a continuous variable. When measured as a categorical variable, the group of participants is divided into the top 30% (i.e., highly sensitive group) and the bottom 70% (i.e., not highly sensitive group; Aron et al., 2012; Pluess et al., 2018). As the SPS scores in the current study were normally distributed, SPS was operationalized as a continuous variable which is in line with the recommendations of Pluess et al. (2018). Notably, higher SPS scores indicate higher levels of sensory processing sensitivity. The Cronbach’s alpha of the questionnaire in our study was 0.76, meeting the criteria of 0.7 for reliable internal consistency.

*Parenting style* was measured using the Parenting Style Inventory-II (PSI-II; Darling and Toyokawa 1997). The scale was designed to assess the construct of parenting styles. Originally, the scale consisted of 36 items with twelve items for each parenting dimension, namely, autonomy-granting, demandingness, and responsiveness. In the current study, a shortened version of the questionnaire was used, with four items each to measure autonomy-granting and demandingness, and three items to measure responsiveness. An example of an item measuring the dimension responsiveness was ‘My parents (or caregivers) are there for me if I have a problem.’ Participants could answer on a 5-point Likert scale (1 = I don’t agree at all, 5 = I completely agree). The parenting dimensions were then used to compute a total score for each of the four parenting styles. For example, a high score for authoritarian parenting represents low responsiveness and autonomy granting and high demandingness. Cronbach’s alpha for the authoritative, authoritarian, permissive and neglectful parenting styles were 0.71, 0.74, 0.71 and 0.75 respectively.

*Internalizing problems* were measured using the Patient Health Questionnaire-4 (*PHQ-4*; Kroenke et al., 2009), an ultra-brief tool for identifying individuals with anxiety and/or depression symptoms. The scale showed good psychometric properties regarding internal reliability as well as construct, factorial, criterion, and process validity despite its limited number of items (Kroenke et al., 2009). The PHQ is suitable for measuring internalizing problems among children and adolescents (López-Torres et al., 2019). The PHQ consisted of two core anxiety items and two core depression items. Examples for items were ‘Over the last 2 weeks, how often have you been bothered by feeling nervous, anxious, or on edge?’ (anxiety) or ‘Over the last 2 weeks, how often have you been bothered by feeling down, depressed, or hopeless?’ (depression). Participants answered on a 4-point Likert scale (1 = Not at all, 4 = Nearly every day), with higher scores indicating higher levels of internalizing problems. For the purpose of this study, the score of all four items were combined to form a total mean score, indicating the general level of internalizing problems the participants are experiencing. Cronbach’s alpha of the total score was 0.71, meeting the criteria of 0.7 for internal consistency.

The *perceived COVID-19 impact* was measured with items from a questionnaire constructed by researchers from the faculty of social sciences at Utrecht University. The COVID-19 questionnaire measured the impact of the COVID-19 pandemic on different areas of life like activity, school, sleep, or atmosphere in the home. Items were for example ‘The COVID-19 crisis has led to more fighting in our family’, ‘I have problems sleeping because of the COVID-19 crisis’ or ‘I worry more about my schoolwork because of the COVID-19 crisis.’ The original questionnaire included 11 items, of which 8 items were selected as most suitable to measure the perceived COVID-19 impact. The items were rated on a 5-point Likert scale ranging from ‘completely disagree’ (=1) to ‘completely agree’ (=5). The scores of all items were combined to form a total mean score, indicating the level of negative COVID-19 impact the participants were perceiving. The Cronbach’s alpha of the questionnaire was 0.57, which is considered a poor internal consistency. However, as the internal consistency is not unacceptable, the questionnaire was included in the current study while keeping the low internal consistency in mind as a limiting factor.

### Data Analyses

For conducting the data analyses, the statistical program SPSS with the PROCESS macro was used. The data-analytic strategy included a stepwise approach: First, a Pearson correlation analysis was conducted to investigate the intercorrelations between SPS, internalizing problems, perceived COVID-19 impact, and the four different parenting styles. In the next step, the mediation model of the perceived COVID-19 impact was investigated using the SPSS macro PROCESS, Model 4 (Hayes, 2017). The moderating effect of parenting style on the relationship between SPS and internalizing problems was tested using the SPSS macro PROCESS, Model 1 (Hayes, 2017). Then the moderated mediation model of perceived COVID-19 impact and parenting style was investigated using the SPSS macro PROCESS, Model 7 (Hayes, 2017). The 95% bias-corrected confidence intervals of the conditional direct and indirect effects were estimated via bootstrapping. The effects were considered significant when the confidence intervals do not include zero. Gender and age are included as control variables. Furthermore, the days in lockdown at the time of data collection was added as a control variable. The lockdown started mid-March 2020 and the data was collected from April until mid-July. In the course of this time, the acuteness of the situation as well as the sort of safety measures in place changed. For participants who filled in the questionnaire beginning of April the COVID-19 situation was still new and safety measures were very strict. Youth that participated at a later point had a chance to get used to the new circumstances but on the other hand were already exposed longer to the COVID-19 restrictions. Hence, prolonged exposure to COVID-restrictions and thus the time of data collection might have affected the perceived COVID-19 impact as reported by the participants.

## Results

### Descriptive Analyses

The descriptive statistics and correlations between the variables of interest are presented in Table 1. The results showed that sensory processing sensitivity (SPS) was positively related to perceived COVID-19 impact and internalizing problems. These findings suggest that a high level of the SPS trait in adolescents is a potential risk factor for perceiving a stronger impact of the COVID-19 pandemic and more internalizing problems. In addition, authoritative and permissive parenting reported by the child were both negatively related to perceived COVID-19 impact and internalizing problems, whereas authoritarian parenting and neglectful parenting were positively related to those variables. Finally, the perceived COVID-19 impact was positively related to internalizing problems.

**Table 1:** Means (M), standard deviations (SD), and correlations of SPS, internalizing problems, perceived COVID-19 impact, and the parenting styles

	*M*	*SD*	1	2	3	4	5	6	7
1. SPS	3.03	0.71	1	–	–	–	–	–	–
2. INTproblems	1.55	0.54	0.372*	1	–	–	–	–	
3. COVID-19	2.33	0.49	0.284*	0.475*	1	–	–	–	–
4. Authoritative	3.96	0.39	−0.053	−0.134*	−0.256*	1	–	–	–
5. Authoritarian	2.51	0.35	0.189*	0.144*	0.373*	−0.565*	1	–	–
6. Neglectful	2.07	0.42	−0.049	0.136*	0.139*	−0.820*	−0.114*	1	–
7. Permissive	3.20	0.32	−0.159*	−0.165*	−0.332*	0.129*	−0.784*	0.072	1

*N* = 395. COVID-19 = perceived COVID-19 impact. Authoritative/Authoritarian/Neglectful/Permissive refer to the respective parenting style

*INTproblems* internalizing problems

*p < 0.01

### Main Effect and Mediation via Perceived COVID-19 Impact

Firstly, the main relationship between SPS and internalizing problems was tested. A regression analyses revealed that the total effect of SPS on internalizing problems in the absence of the mediator (perceived COVID-19 impact) was significant (*β* = 0.37, *p* < 0.001). This supports the hypothesis, that there is a positive relationship between the SPS trait and internalizing problems in adolescents during the lockdown.

**Fig. 2:**
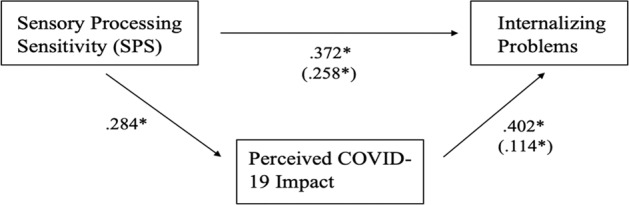
Partial mediation via perceived COVID-19 impact. Note. Relationship between SPS and internalizing problems as mediated by perceived COVID-19 impact. The direct effect of SPS on internalizing problems and the indirect effect via perceived COVID-19 impact are in parentheses. *p < 0.001

Next, the mediation model describing the relationship of SPS with internalizing problems, and the indirect role of perceived COVID-19 impact in this relation (hypotheses 1 and 2) was examined. The analysis was conducted using the PROCESS macro, Model 4 (Hayes, 2017). The results showed a significant positive association between SPS on perceived COVID-19 impact (*β* = 0.284, *p* < 0.001). In the mediation model, the direct effects of SPS on internalizing problems as well as the indirect effect via perceived COVID-19 impact were both significant (*β* = 0.258, *p* < 0.001; *β* = 0.114, *p* < 0.001). The perceived COVID-19 impact partially mediated the association between the SPS trait and internalizing problems (Fig. 2).

### Parenting Style as a Moderator

To examine the moderating effect of parenting style on the relationship between SPS and internalizing problems the PROCESS macro, Model 1 was used (Hayes, 2017). The results of the analyses showed no significant moderating effect for any of the parenting styles (all *p*s > 0.05).

### Moderated Mediation of Parenting Style on SPS and Internalizing Problems via COVID-19 Impact

Analysis of moderated mediation (perceived COVID-19 impact mediates the association between SPS and internalizing problems which in turn is moderated by the parenting style) was conducted using the PROCESS macro, Model 7 (Hayes, 2017). The results for the interaction effect of the different parenting styles are presented in Table 2. No significant moderating effect for any of the parenting styles was found. The results generated by PROCESS examining the moderated mediation as a whole are presented in Table 3. The moderated mediation model was not significant for any parenting style.

**Table 2:** Interaction effect of parenting styles on the relationship between SPS and perceived COVID-19 impact

	Β	*SE*	*t*	*p*
SPS*Authoritative	−0.011	0.075	−0.149	0.882
SPS*Authoritarian	−0.068	0.081	−0.837	0.403
SPS*Permissive	0.021	0.090	0.234	0.815
SPS*Neglectful	0.043	0.066	0.660	0.512

*N* = 395. Bootstrap sample size = 5000

**Table 3:** Moderated mediation of parenting styles as moderators for the mediating effect of perceived COVID-19 impact between SPS and internalizing problems

	Β	*Boot SE*	*BootLLCI*	*BootULCI*
Authoritative	−0.004	0.051	−0.103	0.095
Authoritarian	−0.027	0.030	−0.086	0.034
Permissive	0.008	0.039	−0.073	0.080
Neglectful	0.017	0.039	−0.058	0.095

*N* = 395. Bootstrap sample size = 5000

*LL* low limit, *CI* confidence interval, *UL* upper limit

*p < 0.05

## Discussion

Developmental theories like the diathesis-stress model and the differential susceptibility hypothesis support the notion that children and adolescents respond differently to environmental cues such as stressful life events and the parenting environment, depending on certain personality traits such as sensory processing sensitivity (SPS). The goal of our study was to investigate the relationship between SPS and internalizing problems in youth by testing if the relationship is (partly) mediated by the perceived impact of a stressful life event like the COVID-19 pandemic. Also, it was examined whether this relationship was moderated by parenting style.

In accordance with the first hypothesis, the results of our study show a link between SPS and internalizing problems in adolescents. These findings replicate the results of previous research, which already established the relationship between SPS and internalizing problems, i.e., anxiety and depression (Aron et al., 2005; Bakker & Moulding, 2012; Boterberg & Warreyn, 2016; Dal, 2016; Evers et al., 2008; Liss et al., 2008; Liss et al., 2005). This can be seen as further evidence that high levels of SPS are a risk factor for developing internalizing problems.

Furthermore, the results indicate that the relationship between SPS and internalizing problems is partly mediated by the perceived COVID-19 impact. Partial mediation means that the mediator (here: perceived COVID-19 impact) is only partly responsible for the relationship between SPS and internalizing problems. Even though earlier research demonstrated that the perceived impact of stressful life events can act as a mediator between internalizing problems and associated risk factors (Luby et al., 2006), our study is the first to find this effect for the impact of COVID-19. This goes to show that while it is necessary to give attention to the immediate medical aspects and consequences of COVID-19, it is also imperative to consider the impact the pandemic has had and will continue to have in other areas of life, like the mental health of adolescents.

The finding that the relationship between internalizing problems and SPS is partially and not fully mediated by perceived COVID-19 impact makes further research necessary to understand the precise relationship between SPS and internalizing problems. Future research should focus on identifying alternative mediators and moderators to better understand the pathways that result in problematic outcomes for highly sensitive children. Identifying alternative mediators can open up opportunities for preventive interventions targeting internalizing problems in children with high SPS traits.

Looking at the parenting dimensions, we found that authoritarian and neglectful parenting correlated with worse child outcomes, namely stronger perceived COVID-19 impact and more internalizing problems. As would be expected, the opposite was true for the authoritative parenting style, which is broadly accepted to produce the best child outcomes (Pinquart, 2017). Surprisingly, we also found a negative link between a permissive parenting style and perceived COVID-19 impact as well as internalizing problems. This contrasts with previous studies, which found a positive link between permissive parenting and internalizing problems (Rose et al., 2018). An explanation could be that permissive parenting, which is characterized by low demandingness and high responsiveness, might produce better child outcomes in the unique context of a stressful life event like the COVID-19 pandemic. It is possible that when extraordinary circumstances demand increased flexibility from children and adolescents, the necessity of parental demandingness decreases. Another explanation could be that more permissive parents were not as strict with their children about following the lockdown requirements and for example allowed their children to still see their friends. Not following the rules could potentially buffer against mental health deterioration in the pandemic, albeit in an unrecommended and unsafe way.

Contrary to our expectations, the link between SPS and internalizing problems was not moderated by parenting style. A moderation effect would have meant that the relationship between SPS and internalizing problems depends on a third variable, in this case the parenting style. Finding a moderation effect would have supported the differential susceptibility theory of SPS. According to this theory, individuals scoring high on the SPS personality trait are more susceptible to environmental influences in a for-better-and-for-worse manner, resulting in worse developmental outcomes under negative circumstances but also better developmental outcomes in a supportive environment (Belsky & Pluess, 2016). With regards to parenting, this theory was supported by highly sensitive children showing stronger positive outcomes in relation to positive parenting practices while also showing more negative outcomes as a result of negative parenting practices in earlier research (Liss et al., 2005; Slagt et al., 2018). There are two possible explanations why we did not find the interaction between parenting and sensitivity.

The first explanation is that the moderation effect of parenting does exist but was not found due to methodological shortcomings. Our sample was homogeneous with almost all participants reporting medium to high scores (less than 2% scored lower than 3 and 55% scored 4 or higher on a scale from 1 to 5) on authoritative parenting. The generally positive parenting practices in the sample might have prevented finding a moderating effect.

The second explanation is that SPS may interact with parenting practices, yet not with parenting style as such. Earlier research has established the moderating effect of parenting on SPS for certain aspects of parenting, like parental care, responsiveness, autonomy granting, positive interactions, and inductive discipline (Liss et al., 2005; Slagt et al., 2018). For other aspects of parenting, like parental overprotection, the interaction effect was not found (Liss et al., 2005). It is possible that parenting styles are an aspect of parenting that is too broad to interact with youth sensitivity. In recent years, research has gradually moved from using the global concept of parenting styles to a more specific approach; distinct parenting dimensions like psychological control and adolescent disclosure as well as models looking at domain-specific parenting are on the rise and potentially reflect a more naturalistic picture of the parenting situation (Smetana, 2017). Future research should therefore examine the moderating effect of specific parenting dimensions on the relationship between SPS and internalizing problems among adolescents.

One of the strengths of our study is the large sample with around 400 participants. The sample size allowed for a precise estimation of effect sizes and provided sufficiently reliable results with sufficient precision and power. Another strength is that the data were collected during the lockdown, specifically when lock down restrictions were in place. Data collection during this unique period provided the opportunity to measure the impact of a sudden occurrence of a stressful life event on a larger population and thus testing of the differentially susceptibility model for high SPS children.

The results of our research should be considered in light of several limitations. Firstly, the study had a cross-sectional design. Subsequently, no causal claims can be made on the nature of the relationships between the variables. For example, the correlation between SPS and internalizing problems is in line with the concept of SPS as a risk factor for internalizing problems among adolescents, but a longitudinal study design is necessary to confirm the direction of effect. Secondly, our dataset does not include data on possible atypical development of our participants. As SPS might correlate with neurological issues in development, it would have been useful to include indices for atypical development as a control variable. Thirdly, the internal consistency of the questionnaire measuring the COVID-19 impact turned out to be poor (*α* = 0.57). Under normal circumstances, a questionnaire with such a restricted Cronbach’s Alpha would be revised, as it can be a sign that the items are not measuring the same concept which would impact the reliability of the instrument of measurement. However, the fast pace in which the COVID-19 crisis developed, and the uniqueness of the variable perceived COVID-19 impact did not allow the researchers to either depend on already tested questionnaires or conduct the in-depth analysis that normally accompanies the development of a new questionnaire. As the internal consistency of the COVID-19 questionnaire was not unacceptable, we chosen to use the questionnaire while keeping the low internal consistency in mind as a limiting factor. Lastly, all questionnaires were self-report measures, which holds the risk that the participating youth were answering in a socially desirable manner.

In conclusion, our study shows that high levels of the SPS trait are related to more internalizing problems among adolescents. This main effect is partly mediated by the perceived impact of a stressful life event, the COVID-19 crisis. Future research should focus on identifying more environmental factors that mediate or moderate the relationship between SPS and internalizing problems. By learning more about what environment highly sensitive adolescents need to thrive, we might be able to support sensitive youth and prevent the development of internalizing problems.
